# Optoelectronic crystal of artificial atoms in strain-textured molybdenum disulphide

**DOI:** 10.1038/ncomms8381

**Published:** 2015-06-19

**Authors:** Hong Li, Alex W. Contryman, Xiaofeng Qian, Sina Moeini Ardakani, Yongji Gong, Xingli Wang, Jeffery M. Weisse, Chi Hwan Lee, Jiheng Zhao, Pulickel M. Ajayan, Ju Li, Hari C. Manoharan, Xiaolin Zheng

**Affiliations:** 1 Department of Mechanical Engineering, Stanford University, Stanford, California 94305, USA; 2 Department of Applied Physics, Stanford University, Stanford, California 94305, USA; 3 Department of Materials Science and Engineering, Dwight Look College of Engineering, Texas A&M University, College Station, Texas 77843, USA; 4 Department of Civil and Environmental Engineering, Massachusetts Institute of Technology, Cambridge, Massachusetts 02139, USA; 5 Department of Materials Science & NanoEngineering, Rice University, Houston, Texas 77251, USA; 6 Department of Nuclear Science and Engineering and Department of Materials Science and Engineering, Massachusetts Institute of Technology, Cambridge, Massachusetts 02139, USA; 7 Department of Physics, Stanford University, Stanford, California 94305, USA

## Abstract

The isolation of the two-dimensional semiconductor molybdenum disulphide introduced a new optically active material possessing a band gap that can be facilely tuned via elastic strain. As an atomically thin membrane with exceptional strength, monolayer molybdenum disulphide subjected to biaxial strain can embed wide band gap variations overlapping the visible light spectrum, with calculations showing the modified electronic potential emanating from point-induced tensile strain perturbations mimics the Coulomb potential in a mesoscopic atom. Here we realize and confirm this ‘artificial atom' concept via capillary-pressure-induced nanoindentation of monolayer molybdenum disulphide from a tailored nanopattern, and demonstrate that a synthetic superlattice of these building blocks forms an optoelectronic crystal capable of broadband light absorption and efficient funnelling of photogenerated excitons to points of maximum strain at the artificial-atom nuclei. Such two-dimensional semiconductors with spatially textured band gaps represent a new class of materials, which may find applications in next-generation optoelectronics or photovoltaics.

## Abstract (web summary)

Periodically strained monolayer molybdenum disulphide funnels photogenerated electron-hole pairs across an electric field induced by a spatially graded band gap, mimicking a crystal of artificial atoms. Here, the authors experimentally demonstrate the artificial crystal in strain-textured molybdenum disulphide.

## Graphical abstract

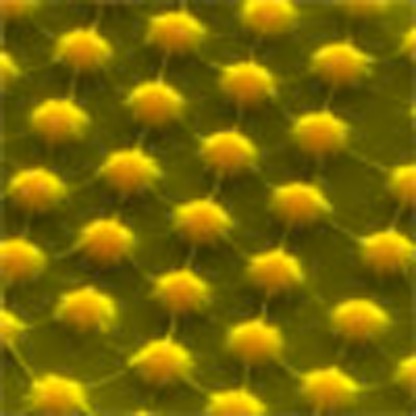

Straining two-dimensional (2D) materials^1^,^2^,^3^,^4^,^5^,^6^,^7^,^8^ with a spatially varying ‘designer' strain can lead to new artificial materials with exotic properties. Assembling such tuned artificial materials in condensed matter is an emerging field and has employed tools such as atomic manipulation^9^ and lithographic techniques^10^. Here we focus on monolayer molybdenum disulphide (MoS_2_), a direct band gap semiconductor that shows promise for applications in photonics and optoelectronics due to its extraordinary physical properties^11^,^12^,^13^,^14^,^15^,^16^,^17^,^18^. Elastic strain offers a novel and exciting opportunity to tune the band gap of monolayer MoS_2_ (refs 1, 2, 3, 4) since it can sustain very high elastic strain before rupturing compared with its bulk counterpart^19^,^20^. For instance, uniaxial strain has been shown to modulate the electronic structure^5^,^6^,^7^ and reduce the optical band gap (OBG) of monolayer MoS_2_ up to 100 meV^6^.

Besides pushing the magnitude of the strain, the ability to apply a spatially controllable strain is even more crucial because it enables the realization of a graded band gap semiconductor eagerly sought for wider photonic, optoelectronic and photovoltaic applications^21^,^22^. It has been calculated that indenting monolayer MoS_2_ creates a tensile strain field that reduces the local quasiparticle band gap (QBG). The resulting modified electronic potential falls off inversely with distance from the indentation, playing the role of an effective electronic potential centred on a mesoscopic ‘artificial atom'^8^. This electronic potential funnels photogenerated excitons from larger band gap regions into smaller band gap regions, resulting in potential broader spectrum light absorption and more efficient photocarrier concentration. The exciton funnel concept has been successfully employed to interpret the OBG of wrinkled few-layer MoS_2_. However, the indirect band gap of few-layer MoS_2_ prevents the distinctive photoluminescence (PL) intensity enhancement expected from the exciton funnelling process from being directly observed^23^. Assembly of the aforementioned artificial atoms would result in a large-area functional ‘artificial crystal.' Currently there exists no fundamental proof-of-principle of this idea, neither at the single ‘artificial atom' level nor at a more practical macroscopic scale.

In the following, we apply spatially modulated biaxial tensile strain in monolayer MoS_2_ using a patterned nanocone substrate to realize the optoelectronic crystal consisting of ‘artificial atoms.' MoS_2_ on top of the nanocones experiences high strain, which gradually decreases from the tip to the perimeter of the nanocones (Fig. 1a). Since the band gap decreases with increasing tensile strain, the nanocone apex marks the ‘nucleus' of the ‘artificial atom'. The strain profile has been optimized for maximum exciton solar funnel collection by controlling the geometry and dimensions of the nanocones.

## Results

### Creation of the artificial-atom crystal in strain-textured MoS_2_

**Figure 1:**
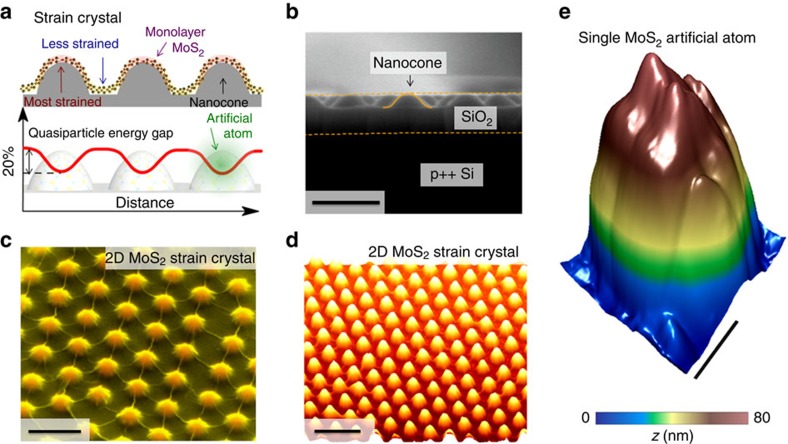
Assembly of an artificial-atom crystal via spatially patterned biaxial strain within a monolayer of MoS_2_. (**a**) Schematic of strained MoS_2_ indented by SiO_2_ nanocones, where the regions on the tips of nanocones exhibit highest tensile strain while the areas between nanocones are less strained. The MoS_2_ energy band gap inversely tracks the strain profile and becomes spatially modulated, forming ‘artificial atoms' at the points of peak strain where the strain-induced potential mimics the Coulomb potential around ions in a crystal. (**b**) Cross-sectional scanning electron microscopy (SEM) image of the nanocone substrate. Dotted lines label the SiO_2_/p++ Si and SiO_2_/vacuum boundaries. Solid curve delineates the nanocone shape. Scale bar is 500 nm. (**c**) Tilted false-colour SEM image of the 2D strained MoS_2_ crystal defined by the nanocone array. Scale bar is 500 nm. (**d**) AFM topography of the 2D MoS_2_ strain crystal. Scale bar is 1 μm. (**e**) STM topography of a single ‘artificial atom' building block within the crystal. Scale bar is 100 nm.

The as-grown MoS_2_ sheet (Supplementary Fig. 1) is transferred onto the SiO_2_ nanocones (Fig. 1b) assembled using nanosphere lithography^24^,^25^ (Supplementary Fig. 2). The as-transferred MoS_2_ is soaked in ethylene glycol to remove the trapped air bubbles and optimize the strain (Supplementary Fig. 3b). The evaporation of ethylene glycol that fills the gap between the MoS_2_ and nanocones generates capillary force that pulls down the MoS_2_ sheet, causing it to conform to the topography of the nanocones and accomplishing the nanoindentation. The clearly visible wrinkles between nanocones (Fig. 1c) indicate the presence of deformation and strain in the MoS_2_ sheet, which is also verified by atomic force microscopy (AFM; Fig. 1d). A scanning tunnelling microscopy (STM) topograph (Fig. 1e) shows the details of a single MoS_2_ artificial-atom element. We note that a similar attempt for templating strain has been carried out in graphene with limited strain magnitude due to a not-yet optimized process^25^,^26^.

### Scanning Raman spectroscopy of the artificial-atom crystal

**Figure 2:**
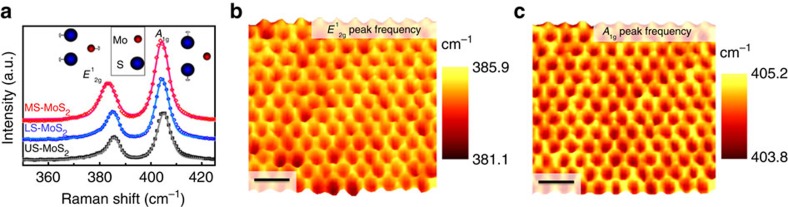
Scanning Raman spectroscopy of the MoS_2_ strain crystal. (**a**) Raman spectra of most strained-MoS_2_, less strained-MoS_2_ and unstrained-MoS_2_. Symbols are measurement data; curves are fitting data. Inset: schematic atomic displacement of the in-plane 
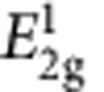
 and out-of-plane *A*_1g_ modes. (**b**,**c**) Scanning Raman spectroscopic maps plotting (**b**) 
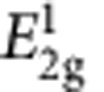
 peak frequency and (**c**) *A*_1g_ peak frequency of strain-textured MoS_2_ on the nanocone substrate. Scale bars are 1 μm.

The spatially varying strain distribution is verified by micro-Raman spectroscopy. Figure 2a shows the typical Raman spectra of the most strained-MoS_2_ (on tip of nanocones), less strained-MoS_2_ (between nanocones) and unstrained-MoS_2_ (on flat SiO_2_ surface) [Supplementary Fig. 4]. The unstrained-MoS_2_ has the typical Raman spectrum of monolayer MoS_2_ with two dominant peaks at 385.6 (
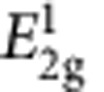
) and 404.9 cm^−1^ (*A*_1g_)^27^,^28^. The strained MoS_2_ samples show significant redshifts for both 
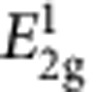
 and *A*_1g_ peaks: 0.8 and 0.3 cm^−1^ for less strained-MoS_2_, and 2.4 and 0.6 cm^−1^ for most strained-MoS_2_. From an overall fit to the redshift magnitudes and the unstrained values, we estimate that (0.230±0.035)% and (0.565±0.025)% biaxial tensile strains are sampled on average by the 450-nm diameter laser beam in less strained-MoS_2_ and most strained-MoS_2_ according to our theoretical calculation discussed later. The Raman mappings of the 
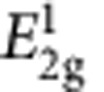
 (Fig. 2b) and *A*_1g_ (Fig. 2c) peak frequencies show that the periodically varying strain is consistent over the entire scanned artificial crystal (25 μm^2^). The darker (lower frequency) and brighter (higher frequency) colours (Fig. 2b,c) indicate that tensile strain is highest on the tips of nanocones—at ‘artificial atom' nuclei—and gradually decreases to a minimum towards the perimeters of the nanocones.

### Scanning PL spectroscopy of the artificial-atom crystal

**Figure 3:**
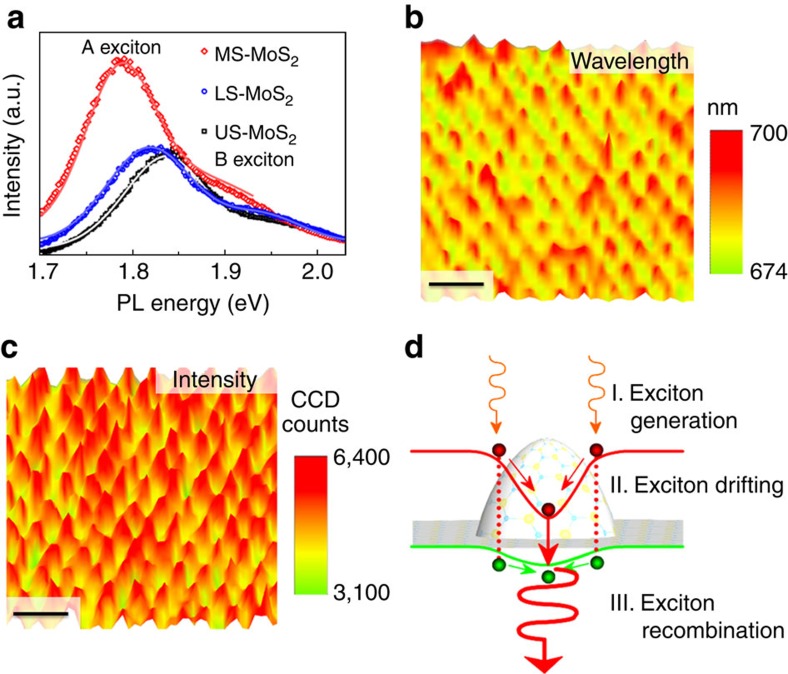
Scanning PL spectroscopy of the MoS_2_ strain crystal. (**a**) PL spectra of most strained-MoS_2_, less strained-MoS_2_ and unstrained-MoS_2_. Symbols are measurement data; curves are fitting data. Scanning PL maps with (**b**) wavelength and (**c**) peak intensity of the same sample and area depicted in Fig. 2. Scale bars are 1 μm. (**d**) Schematic of the funnel effect that consists of three processes: (I) excitons are induced in MoS_2_ upon illumination; (II) photogenerated excitons drift in the artificial atom potential towards the atom center formed by the nanocone tip; and (III) concentrated excitons give emission with longer wavelength.

The effect of strain on the OBG of MoS_2_ strain crystal is examined by micro-PL spectroscopy. Figure 3a compares the representative PL spectra of the most strained-MoS_2_, less strained-MoS_2_, and unstrained-MoS_2_, where the strong PL peaks arise from the direct band gap emissions in monolayer MoS_2_. First, unstrained-MoS_2_ shows a typical PL spectrum of monolayer MoS_2_ with a principal peak at 1.83 eV (A exciton) and a minor peak at 1.95 eV (B exciton)^11^. Second, the strained MoS_2_ clearly exhibits redshift of the A exciton energy: 18 and 50 meV for the less strained-MoS_2_ and most strained-MoS_2_, respectively, indicating a strain-induced OBG reduction^6^,^7^. From the magnitude of the redshift and our theoretical calculation, we estimate approximately 0.2 and 0.5% biaxial tensile strains are optically sampled in less strained-MoS_2_ and most strained-MoS_2_, respectively. These values agree well with the strains derived from Raman peak shifts above. Third, the mapping of the PL wavelength (Fig. 3b) shows that spatially varying OBG reduction is consistently observed over scanned areas. Finally and significantly, the A exciton peak intensity is more than doubled in the most strained-MoS_2_ (Fig. 3a), and the intensity increase is highly reproducible over the scanned region (Fig. 3c). There are a few factors that may affect the PL intensity including strain-modulated oscillator strength, varying local emission geometry, variation in underlying SiO_2_ thickness and strain-induced exciton funnelling^29^. First, according to our exciton calculations (see Discussion below), the change of the oscillator strength on elastic strain is relatively small (∼6% increase under 1% biaxial strain), which cannot explain the observed >100% PL intensity enhancement. Second, the geometry-dependent PL intensity is expected to be enhanced on a flat substrate as the PL intensity of monolayer MoS_2_ peaks at normal collection angle^30^,^31^, which is opposite to our observation. Third, a recent study of PL emission from MoS_2_ shows a flat dependence on underlying SiO_2_ thickness in the range of 180–270 nm, corresponding to oxide thicknesses variations in our samples from etching the nanocones^32^. In fact, the PL emission intensity of homogeneously strained monolayer MoS_2_ was experimentally found to decrease when strain increases due to the increased probability of non-radiative relaxations^6^, again opposite our observations. Finally, we note that enhanced PL intensity due to exciton drifting and concentrating has been observed in strained semiconductor nanowires^29^,^33^,^34^. Therefore, we rule out alternate factors discussed above and attribute the anomalous enhanced PL intensity to the novel 2D exciton funnel effect^8^. The Raman mappings (Fig. 2b,c) show that the tensile strain increases from the perimeters to the tips of the nanocones, so the band gap of MoS_2_ decreases from the perimeters to the tips of the nanocones. Consequently, a built-in electric field pointing from the tip towards the perimeter of the nanocone is created (Fig. 3d) and the photogenerated excitons drift towards the funnel center, as can be modelled analytically and numerically^29^. Since the photogenerated excitons in monolayer MoS_2_ can drift up to 660 nm before recombination^8^, the majority of photogenerated excitons are able to drift from the valleys to the tips of nanocones (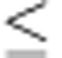
245 nm from pitch radius) without significant recombination. As a result, excitons are concentrated at the tips of nanocones upon illumination and they eventually recombine, giving rise to the enhanced PL emission localized at the nanocone tips (Fig. 3c,d) and supporting the artificial atom and energy funnel concept.

### STM and STS measurements of the artificial-atom crystal

**Figure 4:**
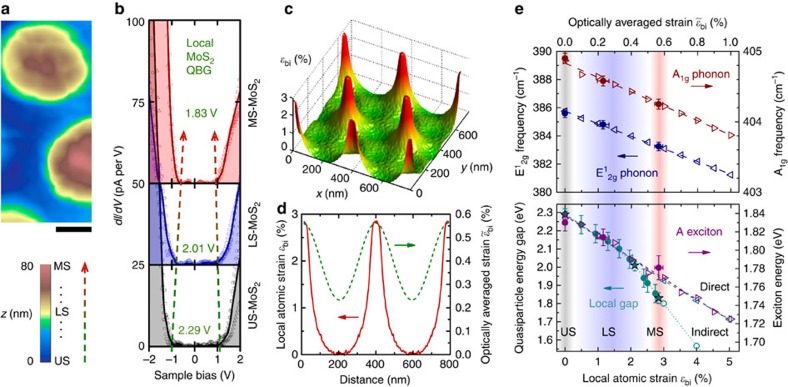
STM/STS of the MoS_2_ strain crystal and experiment–theory correlation. (**a**) STM topography in the vicinity of three nanocones. Height colour bar indicates unstrained (topographic valleys), less strained (intermediate strain) and most strained (topographic peaks) to denote the amount of local strain expected at each height *z* (dashed arrow indicates direction of increasing strain). Scale bar is 100 nm. (**b**) Representative local d*I/*d*V* measurements acquired from the region shown in (**a**) from unstrained (grey), less strained (blue) and most strained (red) locations. Each strain shows three individual d*I/*d*V* measurements (open symbols), which are acquired in tight groups <10 nm in extent over each location. The solid lines are the averages of each group of three curves. Band gaps extracted from the curves are enumerated for each strain amount with the trend indicated by dashed arrows. (**c**) Calculated local biaxial strain distribution 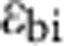 over several unit cells of the crystal. (**d**) Cross-section of the strain profile showing local atomic strain 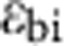 (red), measured by STS and ranging from 0 to 2.85% tensile strain, and the optically averaged strain 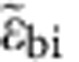 (green) sampled by scanning Raman/PL measurements, ranging from 0.23 to 0.56% tensile strain. The broadened distribution is calculated from theoretical local strain using the 450-nm optical Gaussian beam diameter. (**e**) Top panel shows theoretical (open symbols) and experimental (filled circles) Raman frequencies for the indicated phonon modes as a function of strain. Dashed lines are linear fits to the theory (−1.02 cm^-1^ per % for *A*_1g_ phonon and −4.48 cm^-1^ per % for 
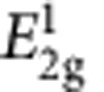
 phonon) and are used for the optically averaged strain assignment (top axis). Bottom panel shows theoretical (open symbols) and experimental (closed symbols) data for the OBG (magenta, right axis) and the local QBG (cyan, left axis). Axes have been scaled using the results of (**d**) as discussed in main text. Dashed lines match fit of −0.11 eV per % (direct OBG) and dotted line shows an increased −0.24 eV per % (indirect OBG) falloff of the OBG beyond 2% biaxial strain. Star symbols denote the three specific measurements shown in (**b**). Error bars are determined by s.d. of each spectroscopic measurement.

The local electronic structure modulation of MoS_2_ caused by strain is directly verified by STM. Figure 4a shows an STM topograph (*V*=2.4 V, *I*=18 pA) in the vicinity of three nanocones with height variations of 80 nm. Scanning tunnelling spectroscopy (STS) is performed in different regions of the area shown, and representative d*I/*d*V* tunnel spectra for unstrained-MoS_2_, less strained-MoS_2_, and most strained-MoS_2_ are shown in Fig. 4b. It is worth noting that these spectra show the Fermi level near mid-gap position due to the electron depletion caused by the gold substrate^35^. The QBG sizes extracted from the d*I/*d*V* spectra (Supplementary Fig. 5) are labelled at each strain level. Unstrained-MoS_2_ shows a QBG of 2.29 eV, which is consistent with the OBG of 1.83 eV obtained by PL measurement considering the exciton binding energy ∼0.5 eV^8^. The extracted local QBG varies from 2.29 eV down to 1.83 eV from STS measurements acquired from unstrained-MoS_2_ (topographic valley), through less strained-MoS_2_ (intermediate regions) and to most strained-MoS_2_ (topographic peak) locations. This corresponds to a measured local strain approaching ∼3% for most strained-MoS_2_. These strains are much higher than the maximum of ∼0.6% measured by Raman spectroscopy, which can be explained by the inherently atomic scale measurement of STM/STS compared with the optical measurements, which average over the finite laser beam size (∼450 nm).

To quantify this argument and verify the complete strain profile of the crystal, we simulate the strain induced in a monolayer MoS_2_ sheet by applying a constant pressure on the sheet, and Lennard–Jones interatomic interactions between the sheet and substrate (Fig. 4c, Supplementary Fig. 6). To calibrate the mapping between the local tunnelling measurements and the optically averaged Raman/PL measurements, we plot in Fig. 4d the cone-to-cone calculated strain profiles (Fig. 4c) before and after a convolution step. The raw atomic strain 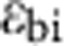 is convolved with a 2D Gaussian of 450-nm 1/*e*^2^ width, corresponding to the 450-nm diameter Gaussian beam used in our optical experiments. With no fitting parameters, this yields a predicted optically averaged strain sampled by scanning Raman/PL; this average strain 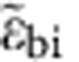 ranges from 0.233 to 0.562% and is in excellent agreement with the Raman/PL results from less strained-MoS_2_ and most strained-MoS_2_ regions presented above. This result provides confirmation that the local atomic strain 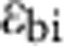 is approaching the unprecedentedly high value of 3%.

## Discussion

We use this calibration to unify all experimental (closed symbols) and theoretical (open symbols) results (Fig. 4e). We calculated Raman spectra under 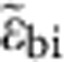 from 0 to 1% (red and blue triangles in Fig. 4e; Supplementary Fig. 7a). As 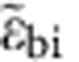 increases from 0 to 1%, the 
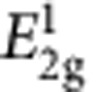
 and *A*_1g_ peaks shift towards lower frequencies. Such Raman peak shifts vary linearly with biaxial strain, and the slopes are −4.48 and −1.02 cm^−1^ per 1% of biaxial strain for 
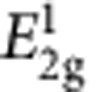
 and *A*_1g_ modes, respectively (dashed red and blue lines in Fig. 4e). The fit to all Raman data in unstrained, less strained and most strained regions with respect to these two lines yields 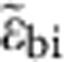 values quoted earlier, (0.230±0.035)% (less strained) and (0.565±0.025)% (most strained) that are shaded in Fig. 4e. The calculated A exciton energy at various 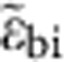 ranging from 0 to 1% is shown in Fig. 4e (lower panel, open purple triangles; Supplementary Fig. 7b). The A exciton peak exhibits a linear redshift rate of −0.11 eV per 1% of biaxial tensile strain (dashed purple line in Fig. 4e, also in good agreement with other calculations^1^,^4^). Our recorded PL energies when assigned to the Raman-extracted strain values above fall along this redshift line within experimental error.

The above discussion correlates OBG measurements to 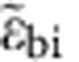. To make the final quantitative link to the intrinsic QBG and the local atomic strain 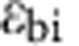 within the artificial crystal, we use the results of the convolution analysis elaborated above. Accordingly, we show in Fig. 4e a local atomic strain axis 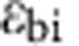, which is linked to the optically averaged strain axis 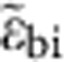 by the ratio of 
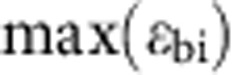
:
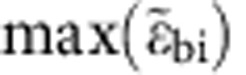
=2.85:0.562 ∼ 5:1. On this axis we first plot the calculated theoretical QBG from 0 to 5% local strain for direct (open cyan triangles) and indirect (open cyan circles) transitions^8^. The fit to the direct gap calculations (Fig. 4e, dashed cyan line) also diminishes at −0.11 eV per % biaxial strain and the gap axis there is scaled by the same 5:1 ratio to show this match. The calculations^8^ furthermore reveal a direct-to-indirect gap transition at ∼2% biaxial tensile strain, resulting in an increased drop of ∼−0.24 eV per % of the (now indirect) gap with strain (Fig. 4e, dotted cyan line). We add other acquired STS data to this plot. The closed cyan star symbols correspond to the curves shown in Fig. 4b and provide the anchor for the strain measurement: the highest QBG of 2.29 eV corresponds to zero local strain, and the lowest QBG of 1.83 eV corresponds to the highest strain at the artificial-atom nanocone tips, and must correspond to the largest optically averaged strains deduced. The remaining STS measurements have known gap values, and strain values that can be inferred by assigning 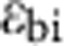 to each value along the dotted line: gaps falling at −0.11 eV per % from the unstrained-MoS_2_ value and rising at 0.24 eV per % from the most strained-MoS_2_ value, meeting at the 2% direct-to-indirect transition. We note that the strain magnitude of 3% is calculated based on biaxial strain. This is the lower bound as larger strain magnitude is necessary to achieve the observed band gap modulation if the strain is uniaxial, which could exist in local areas due to the imperfect geometry of the nanocones.

We note that this summary shows that our local crystal strain 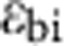 is sufficiently high to access the indirect band gap of monolayer MoS_2_. However, because the Raman/PL measurements are optically averaged, they are dominated by the majority of the points within the laser beam that have direct transition as evidenced by the high PL efficiency. The measurements also confirm a remarkably large exciton binding energy of ∼0.5 eV, only recently verified to be a general feature of dichalcogenides^36^. From the STS measurements, a modulation of over 20% in the QBG is directly observed. From the mapping to the optically averaged strain (Fig. 4) and the deduced exciton binding energy, this represents a huge modulation of the OBG, exceeding 25%.

This engineered OBG enables a broadband light absorption by increasing the absorption bandwidth from 677 nm (unstrained-MoS_2_) to 905 nm (most strained-MoS_2_), which covers the entire visible wavelength and most intensive wavelengths of the solar spectrum. While already without precedent, since these strains are not yet even rupture limited, we anticipate even larger band gap variations and electronic fields can be embedded in such artificial crystals in the future.

## Methods

### Transfer and strain of MoS_2_ monolayer

MoS_2_ monolayers grown by chemical vapour deposition were transferred onto the SiO_2_ nanocone substrate using the PMMA-assisted wet transfer process. The transferred sample was then baked at 100 °C for 30 min and the PMMA was removed by sequential soaking in acetone at 60 °C for 10 min followed by chloroform at 60 °C for 1 h. Afterwards, the SiO_2_ nanocone with transferred MoS_2_ was immersed in ethylene glycol in vacuum for 1 h to ensure that both sides of the MoS_2_ sheet were wetted by ethylene glycol. Lastly, the sample was dried in ambient air to completely evaporate ethylene glycol.

### Raman and photoluminescence characterizations

The Raman and PL measurements were performed with the excitation laser line of 532 nm using a WITEC alpha500 Confocal Raman system in ambient air environment. The power of the excitation laser line was kept below 1 mW to avoid damage of MoS_2_. The Raman scattering was collected by an Olympus 100 × objective (N.A.=0.9) and dispersed by 1,800 (for Raman measurements) and 600 (for PL measurements) lines per mm gratings.

### STM/STS measurements

STM/STS measurements were performed at 77 K in ultrahigh vacuum. A bilayer of titanium (10 nm) and gold (80 nm) films were deposited onto the silicon oxide nanocone surface, then monolayer MoS_2_ flakes were transferred and strained on the metalized nanocone surface. A control sample was prepared alongside the STM sample and characterized using SEM and AFM to ensure conformal coating of MoS_2_ on the metalized nanocones. The STM sample was annealed in the STM ultrahigh vacuum chamber at 200 ^o^C for 1 h to clean the MoS_2_ surface for a reliable STM measurement.

### Modelling of strain distribution of MoS_2_ on nanocones

The MoS_2_ sheet was modelled using honeycomb lattice with Tersoff potential and the substrate was modelled as a fcc lattice. A Lennard–Jones potential was employed to include the Van der Waals interaction between substrate and the MoS_2_ sheet. A constant force was placed on each atom downward to emulate the pressure difference across the MoS_2_ sheet (see Supplementary Fig. 6 for more details).

### Theoretical Raman spectra of monolayer MoS_2_

The theoretical biaxial strain-dependent Raman spectra were calculated using first-principles density-functional perturbation theory implemented in the Quantum-ESPRESSO package with a plane-wave cutoff of 120 Rydberg, a Monkhorst–Pack k-point sampling of 12 × 12 × 1, and an exchange correlation functional of the Perdew–Zunger form within the local density approximation. The spin-orbit coupling was not included. In addition, norm-conserving Hartwigsen–Goedecker–Hutter pseudopotentials were used to take into account the core electrons and reduce the computational efforts. All biaxially strained configuration were fully relaxed with a convergence criteria of 0.0001, a.u. for the maximal residual force. The calculation was carried out in a periodic supercell with a vacuum spacing of 20 Å along the *z* (plane normal) direction to reduce the spurious interaction between the neighbouring unit cells.

### Theoretical optical absorption spectra of monolayer MoS_2_

The theoretical biaxial strain-dependent optical absorption spectra were calculated by solving the Bethe–Salpeter equation within the Tamm–Dancoff approximation. The key parameters used in the Bethe–Salpeter equation including quasiparticle energies and screened-Coulomb interactions calculated were obtained from many-body perturbation theory with the Hedin's GW approximation. All the calculations were performed using the Vienna Ab initio Simulation Package with plane-wave basis and the projector-augmented wave method. A plane-wave cutoff of 350 eV, a Monkhorst–Pack k-point sampling of 18 × 18 × 1, and an exchange correlation functional of the Perdew–Berke–Ernzerhof form within the generalized gradient approximation were used. All biaxially strained configurations were fully relaxed with the maximal residual force of ≤0.0001, eV Å^−1^ using density-functional theory calculations.

## Additional information

**How to cite this article:** Hong, L. *et al.* Optoelectronic crystal of artificial atoms in strain-textured molybdenum disulphide. *Nat. Commun.* 6:7381 doi: 10.1038/ncomms8381 (2015).

## Supplementary Material

Supplementary Information Supplementary Figures 1-8, Supplementary Notes 1-6 and Supplementary References
