# Supplementary material for: Optoelectronic crystal of artificial atoms in strain-textured molybdenum disulphide
Source: Nat Commun. 2015 Jun 19;6:7381. doi: 10.1038/ncomms8381 (PMC4557352; doi:10.1038/ncomms8381)
Supplement: Supplementary Information — Supplementary Figures 1-8, Supplementary Notes 1-6 and Supplementary References [file ncomms8381-s1.pdf]

Supplementary Figures

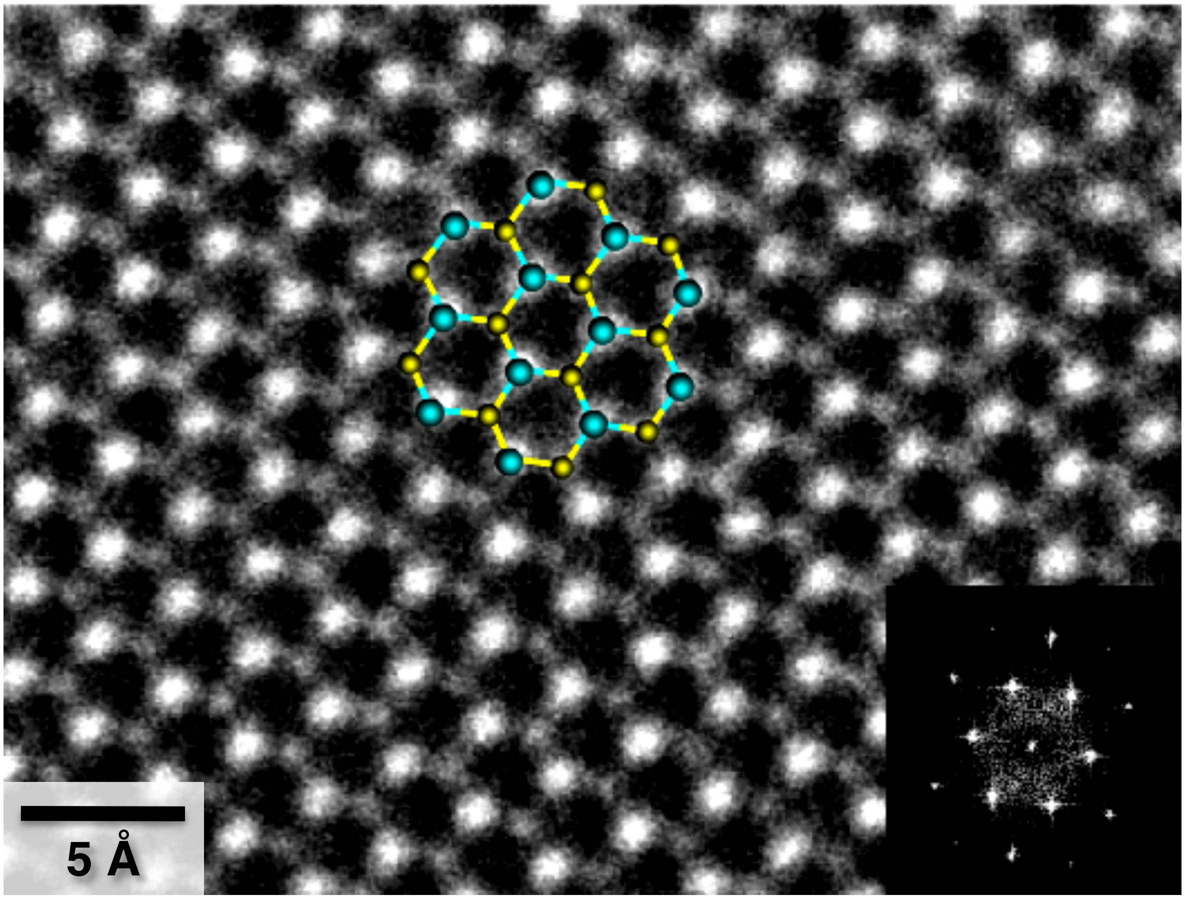

Supplementary Figure 1 | TEM image of CVD-grown monolayer MoS_2. The schematic overlay shows the atomic configuration of MoS_2. Right inset: Fourier transform image of the lattice. Scale bar is 5 Å.

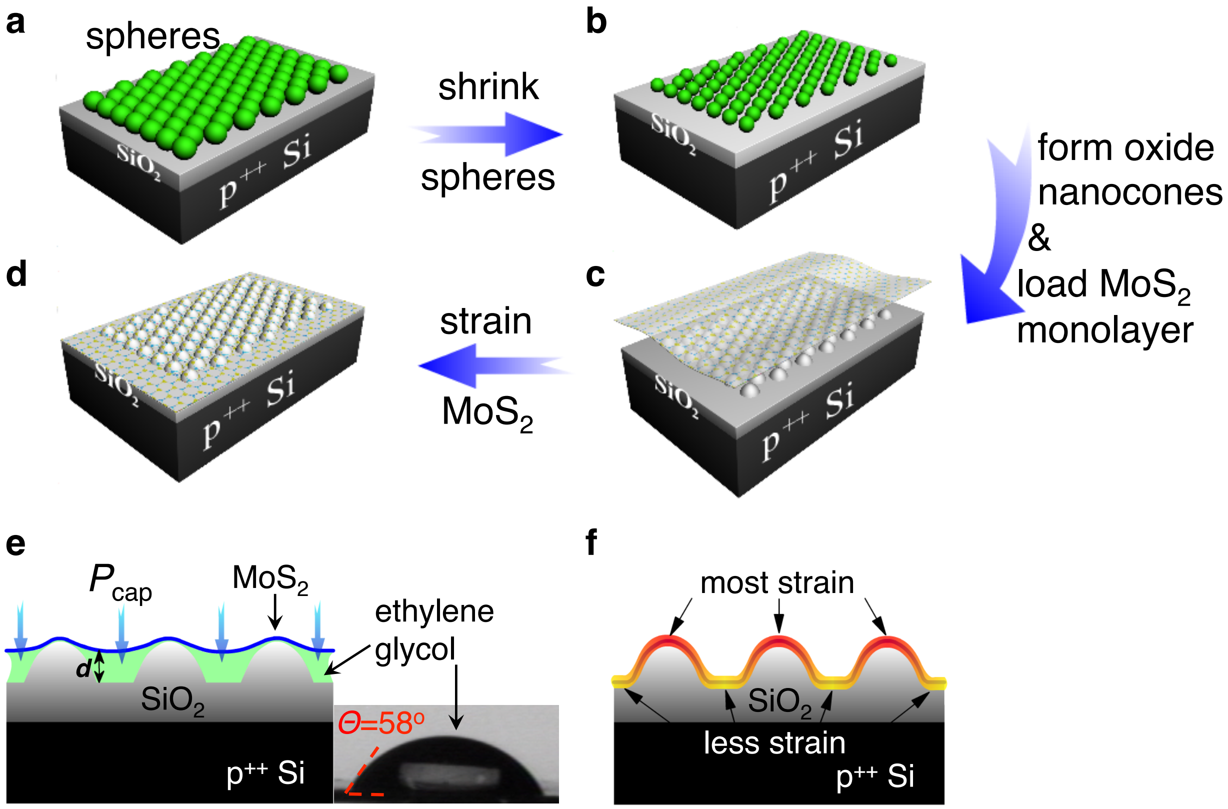

Supplementary Figure 2 | Schematic of the transfer and straining process. a, Monolayer nanospheres are assembled on the SiO_2/Si substrate. **b**, Nanospheres with reduced sizes are used as an etch mask to create nanocones in SiO_2 substrate. **c**, MoS_2 is transferred onto the SiO_2 nanocone array after removing nanosphere mask. Solvent capillary force is applied to strain MoS_2. **d**, MoS_2 conformally coats SiO_2 nanocone surface when solvent completely dries. **e**, Schematic cross-section view of the capillary force that pulls down MoS_2 against the SiO_2 nanocones when solvent evaporates. d is the distance between MoS_2 and substrate. Arrows indicate the force being exerted on MoS_2. Inset, right: a side-view image of an ethylene glycol drop on MoS_2 flakes. $\theta = 58^o$ is the contact angle between ethylene glycol and MoS_2. **f**, Strained MoS_2 on SiO_2 nanocones, where the areas on tip of the nanocones are most strained and the areas between nanocones are less strained.

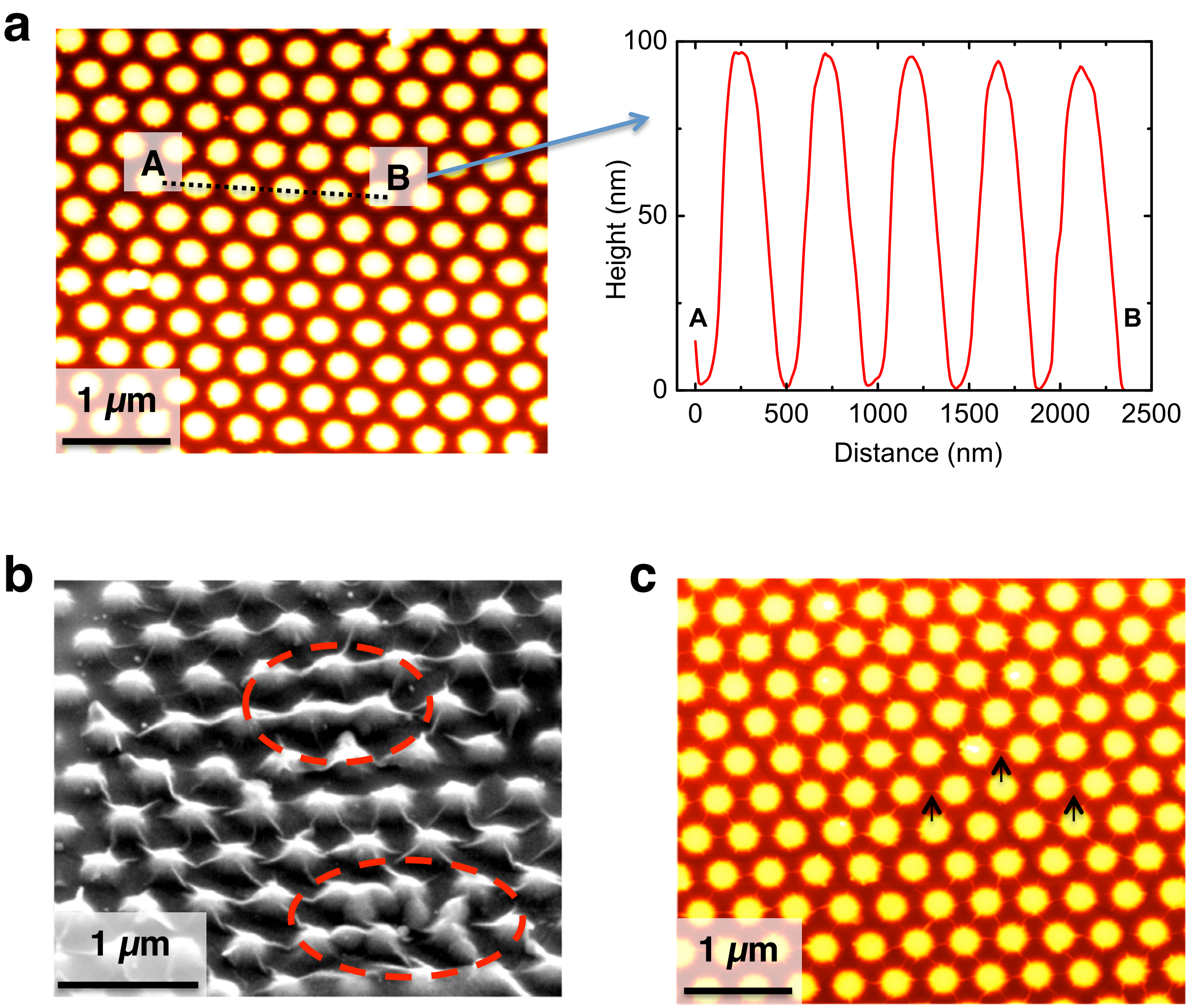

Supplementary Figure 3 | AFM and SEM images. **a**, AFM image of nanocones only before loading the MoS_2 monolayer. Right: the height profile along dotted line A-B. **b**, Tilted SEM image of nanocone array covered by as-transferred MoS_2. Dashed circles indicate air bubbles trapped under the MoS_2 membrane after transfer. **c**, AFM image of the strained MoS_2 membrane on nanocones. Arrows delineate three tiny wrinkles. Scale bars are 1 μm.

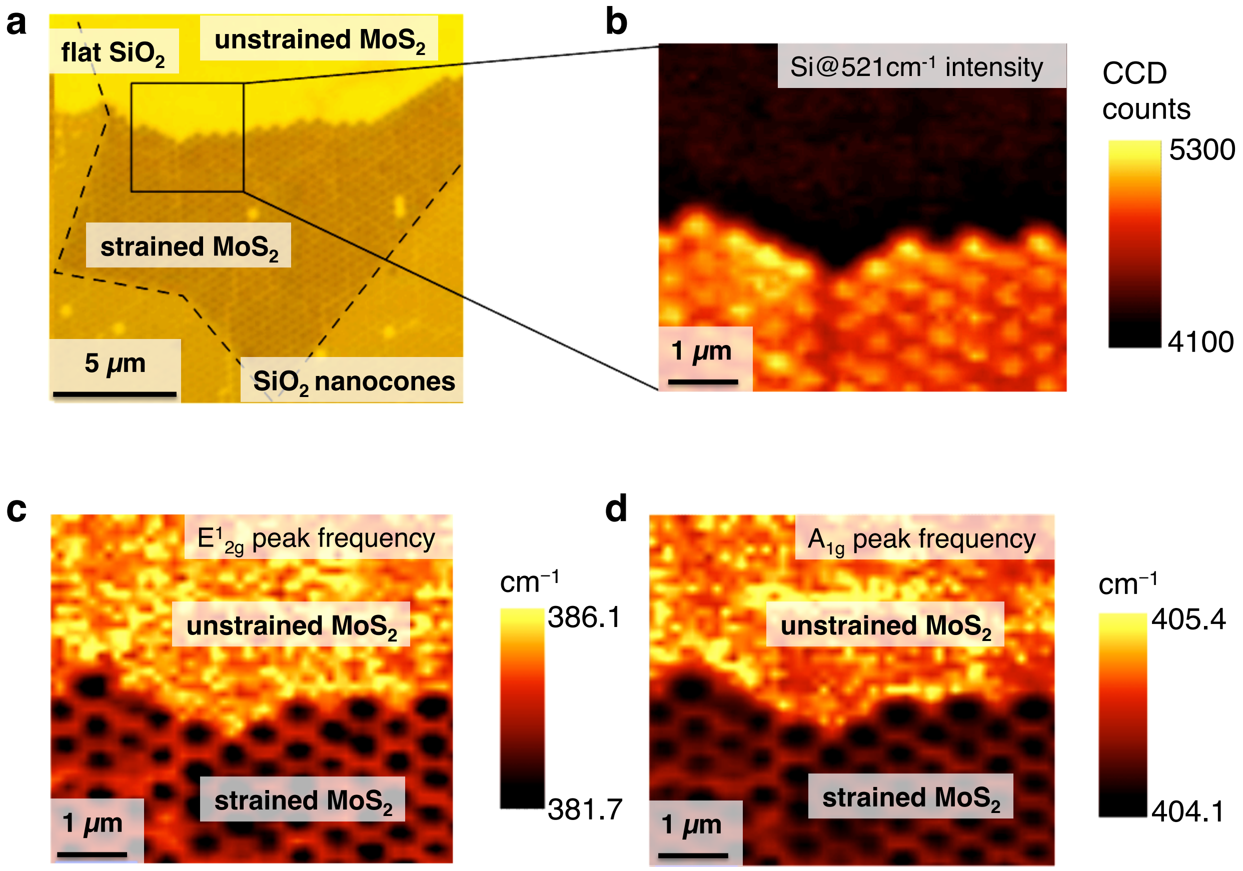

Supplementary Figure 4 | Boundary between unstrained and strained MoS_2. a, Optical microscope image of a MoS_2 flake lying across the boundary between the SiO_2 nanocone region and a flat SiO_2 region. Scale bar is 5 µm. Dashed lines enclose the region of MoS_2. Scanning Raman images with (**b**) peak intensity of silicon at 521 cm^{-1}, **c**, E^1_{2g} peak frequency, and **d** A_{1g} peak frequency of MoS_2 in the square in (**a**). Scale bars are 1 µm.

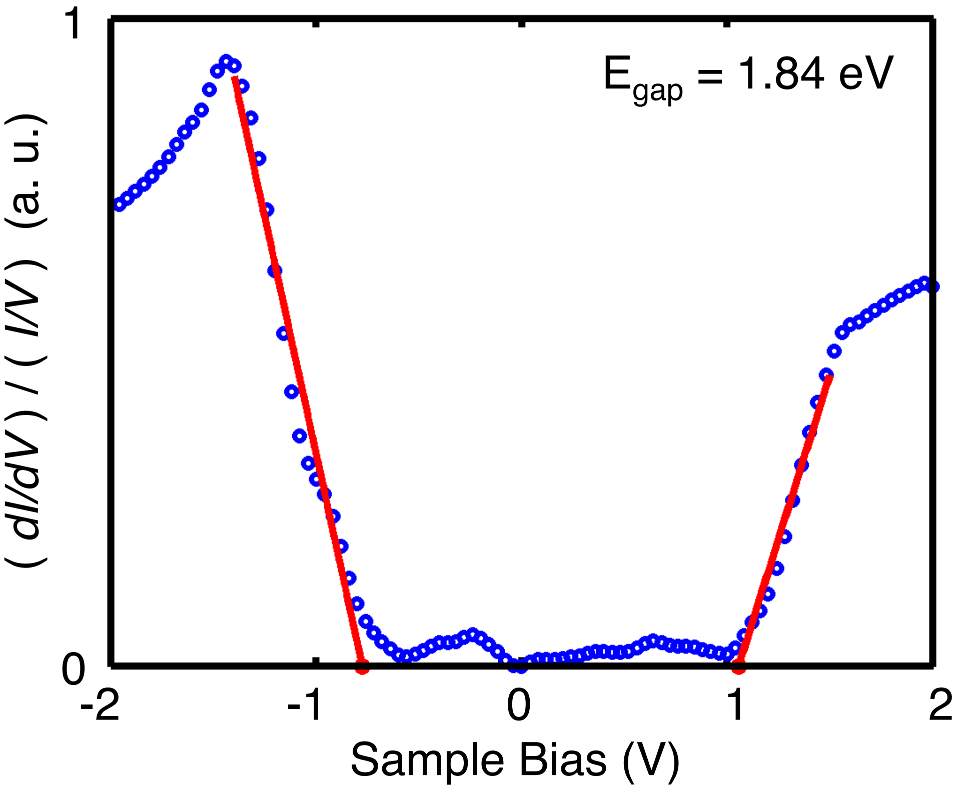

Supplementary Figure 5 | STS quasiparticle band gap fitting method. The blue circles show *dI/dV* data normalized by *I/V*. The red lines are a first order polynomial fit to the conduction and valence bands which are extrapolated to zero to find the band edges, which correspond to a band gap size of 1.84 eV in this example from the MS-MoS_2 area.

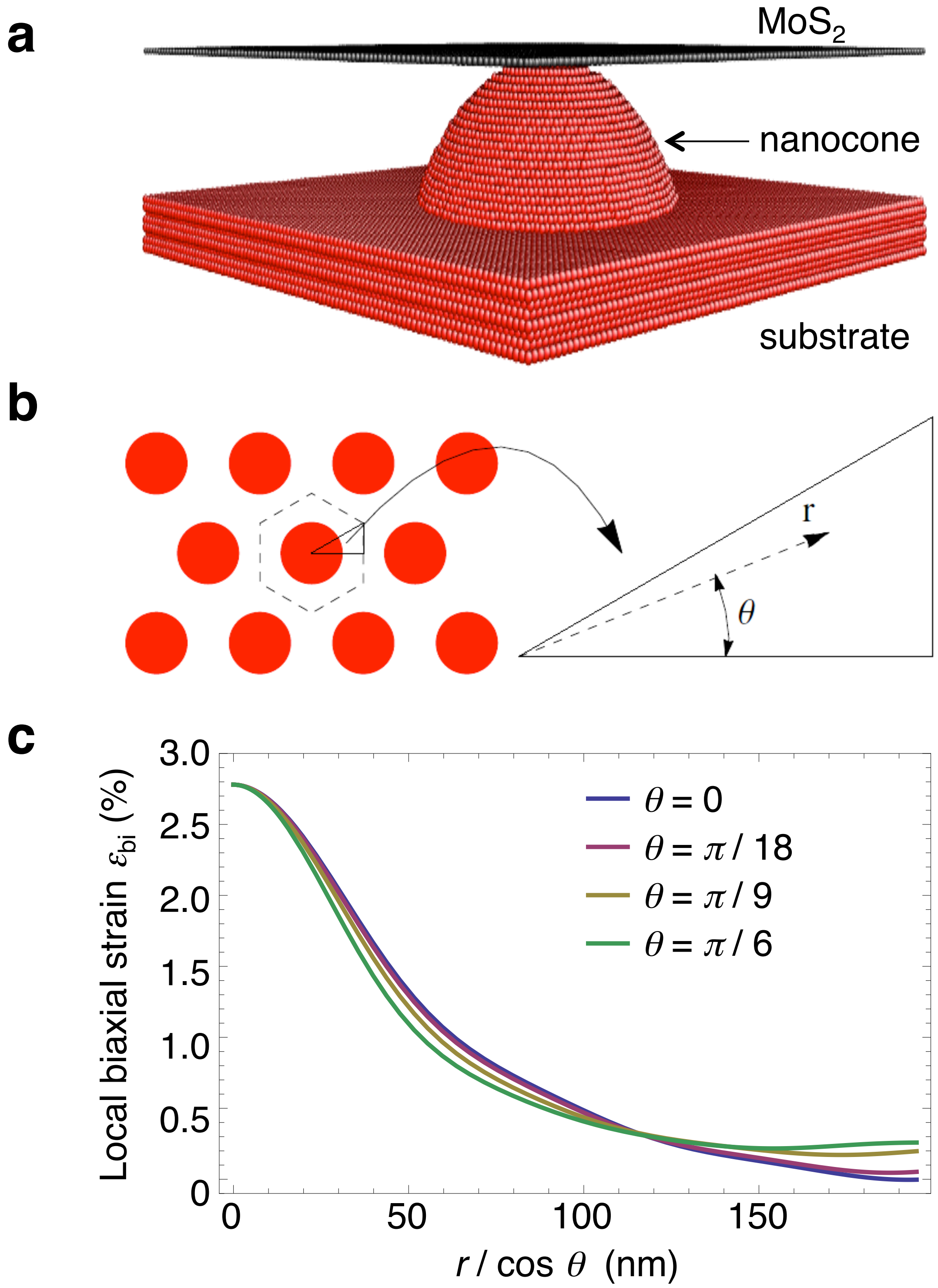

Supplementary Figure 6 | Modeling of the geometry of strained MoS_2. a, A thin sheet with honeycomb lattice (MoS_2) is above the nanocone substrate. The nanocone is 90 nm in height and 250 nm in diameter. **b**, 12-fold symmetry. **c**, Resulting local biaxial strain magnitude versus $r/\cos(\theta)$.

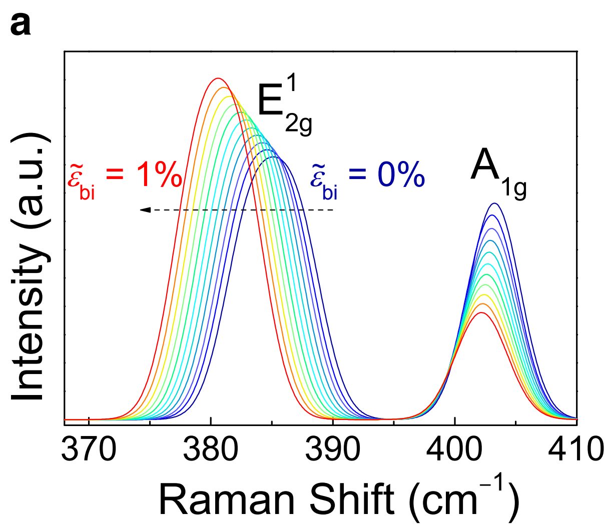

Supplementary Figure 7 | Calculated Raman spectra and optical absorption. a, Calculated Raman spectra under average biaxial strain $\tilde{\varepsilon}_{bi}$ ranging from 0 to 1%. **b**, Calculated optical absorption spectra of MoS_2 under average biaxial strain $\tilde{\varepsilon}_{bi}$ ranging from 0 to 1%.

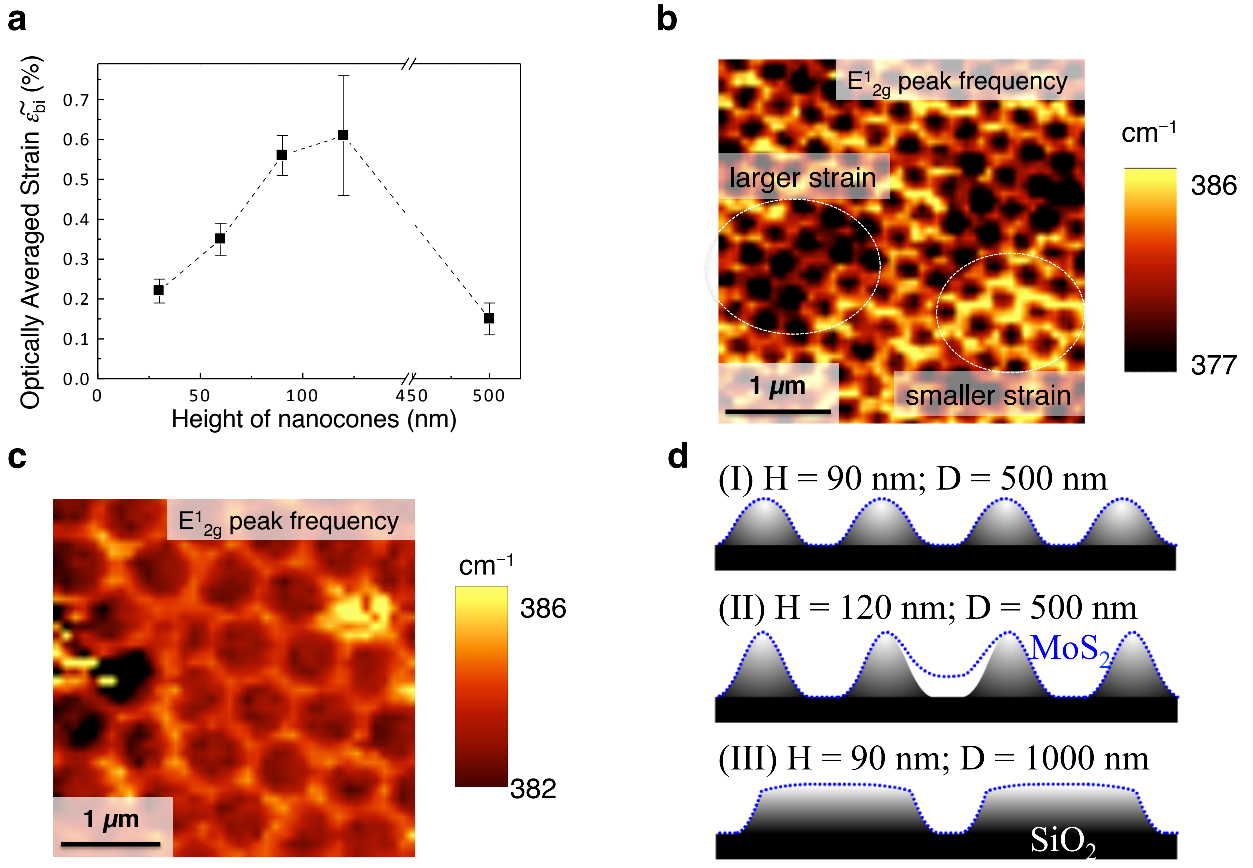

Supplementary Figure 8 | Strained MoS_2 on nanostructured substrate with various geometries and dimensions. **a**, Optically averaged strain versus the height of nanocones with tip-to-tip distance of 500 nm. Scanning Raman images with E^1_{2g} peak frequency of **b**, 120-nm-tall nanocones with tip-to-tip distance of 500 nm, and **c**, 90-nm-tall nanocones with tip-to-tip distance of 1000 nm. Scale bars are 1 μm. Dashed circles label the area of larger and smaller strain, respectively. **d**, Schematic of MoS_2 strained on nanocones with (I) height (H) of 90 nm and tip-to-tip distance (D) of 500 nm, (II) H = 120 nm; D = 500 nm and (III) H = 90 nm; D = 1000 nm. Dotted blue lines represent the MoS_2 sheet.

Supplementary Note 1: MoS_2 growth and characterization

Growth of MoS_2 using chemical vapor deposition (CVD) method

Molybdenum trioxide (MoO_3) and sulfur (S) powder were used as sources. 5 mg MoO_3 powder was loaded in a ceramic boat. A piece of Si wafer capped with 270 nm SiO_2 layer was suspended on the ceramic boat with the polished side facing down. Then the ceramic boat was located at the center of a quartz tube furnace. Another ceramic boat containing sulfur powder (1 g) was placed at an upstream position where the temperature would reach 200 ºC during the growth. After sealing, the tube was purged with 50 sccm argon (Ar) gas for 10 min at room temperature, then heated to 750 ºC within 15 min, and maintained at 750 ºC for 20 min. The system was then allowed to cool down to room temperature naturally[1].

Transmission electron microscopy (TEM) characterization of CVD-grown monolayer MoS_2

The as-grown MoS_2 film was spin coated with 1 μm-thick PMMA film and immersed in 1M potassium peroxide (KOH) solution overnight. After the SiO_2 layer was etched away, the PMMA-MoS_2 film floating on the water was transferred to DI water, and then it was scooped up with a TEM grid. After drying with a small gas flow, the grid was immersed in acetone to remove the PMMA film, followed by IPA and DI water wash. The sample on TEM grid was characterized with a FEI spherical aberration (image)-corrected 80-300 Titan environmental (scanning) (FEI Titan E-(S)TEM) at 80 KV. A high-resolution TEM image is shown in Supplementary Figure 1, where the bright and dim atoms are molybdenum and sulfur atoms, respectively. They are arranged in a perfect hexagonal structure as shown by the schematic atomic structure overlaid on the TEM image. A Fourier transform image is inserted in Supplementary Figure 1, confirming the hexagonal structure of the film as well.

Supplementary Note 2: Straining method

Transfer and straining of MoS_2

Silicon wafers capped with 270 nm thermally grown oxide were soaked in piranha at 100 ^{o}C for 2 hours to achieve a clean and hydrophilic surface. Monolayer polystyrene (PS) or silica nanospheres with a diameter of about 490 nm were assembled on SiO_2 surface by spin coating or

Langmuir Blodgett method (Supplementary Figure 2a). Afterward, the size of the nanospheres was reduced by plasma to serve as an etch mask (Supplementary Figure 2b). Then the substrate was etched in fluoroform (CHF_3) and oxygen plasma with the nanosphere mask. Lastly, the nanosphere mask was removed by sonication in chloroform. The resulting base diameter and height of the SiO_2 nanocones were about 250 nm and 90 nm, respectively. The CVD monolayer MoS_2 sample was coated with a layer of PMMA and soaked in 1M KOH solution at 80 °C. After the PMMA-MoS_2 layer was lifted off in the KOH solution, it was transferred to deionized (DI) water to remove KOH residue. The SiO_2 nanocone substrate was then used to fish the PMMA-MoS_2 bilayer floating on DI water (Supplementary Figure 2c). The sample was then baked at 100 °C for 30 minutes before being soaked in acetone, followed by chloroform to remove PMMA completely. This heating step also effectively removed the water trapped below the PMMA/MoS_2 bilayer film, excluding the influence of water on the optical characterizations. Afterwards, the nanocone patterned SiO_2 with MoS_2 was soaked in ethylene glycol (EG) in vacuum for 1 hour to ensure both sides of MoS_2 membrane were wet by EG. Lastly, the sample was dried in air to evaporate the EG completely (Supplementary Figure 2d). As such, MoS_2 membrane coated the nanocone surface due to capillary force during solvent evaporation (Supplementary Figures 2e-f). We noted that MoS_2 can be directly grown on top of the nanocones to achieve the conformal coating, but non-uniform multilayer MoS_2 flakes were usually grown on nanocones. The poor growth was likely due to that the etched SiO_2 nanocone surface has many nucleation sites. This is one of the motivations for developing the transfer-strain process.

Contact angle measurement of ethylene glycol on MoS_2

As the CVD-grown monolayer MoS_2 was not a continuous film, natural MoS_2 flake (purchased from SPI supplies) were used to measure the contact angle between EG and MoS_2. 100 μL of EG solution was dropped onto MoS_2 flake surface using a micropipette. The side-view image of the droplet was taken, as shown in Supplementary Figure 2e (inset). A contact angle of about 58° was obtained.

Estimation of capillary pressure

A noticeable feature of our straining method is the utilization of capillary force[2,3] to generate biaxial tensile strain in monolayer MoS_2. The magnitude of the capillary pressure can be estimated from $P_{\mathrm{cap}} = 2\gamma\cos(\theta)/d$, where γ is the surface tension of the solvent, θ is the contact angle between the solvent and MoS_2 sheet, and d is the distance between MoS_2 sheet and substrate[3]. We used EG as the solvent since it has a large surface tension about 48 mN/m at room temperature[4], as well as a good wettability with the MoS_2 surface (the contact angle between MoS_2 flake and EG is 58° as shown in Supplementary Figure 2e, inset). Since the SiO_2 nanocone is about 90 nm in height, the estimated capillary pressure P_{cap} is about 5×10^5 Pa. The amount of pressure required to pull the center of MoS_2 sheet between two nanocones to contact the substrate can be estimated from $P_{\mathrm{def}} = 64Et^3Z_{\mathrm{c}}/W^4$, where $E \approx 270$ GPa is the Young's modulus of monolayer MoS_2, $t = 0.67$ nm is monolayer MoS_2 thickness, $W = 490$ nm is the width of the membrane (nanocone tip-to-tip distance), and $Z_{\mathrm{c}} \cong 90$ nm is the amount of vertical deformation. The required pressure is estimated to be $P_{\mathrm{def}} = 1\times10^4$ Pa, which is much smaller than the capillary pressure P_{cap} that can be generated by EG evaporation. In other words, the capillary force is sufficient to deform the MoS_2 to contact the nanocone surface.

Supplementary Note 3: Comparison between strained and unstrained MoS_2

The AFM image of the nanocone substrate is shown in Supplementary Figure 3a, and the height profile along line A-B is inset. The height and width of the nanocone are around 90 and 250 nm, respectively. From the tiled SEM image of MoS_2 on nanocone (Supplementary Figure 3b), one can see that the as-transferred MoS_2 is partially strained due to acetone drying process during PMMA removal. However, many air bubbles trapped below the MoS_2 sheet can be seen, as labeled by the dashed circles. After straining MoS_2 on nanocones with EG, complete straining of MoS_2 is achieved. Wrinkles due to MoS_2 deformation appear between nanocones, as shown in Supplementary Figure 3c.

The optical microcopy image of the boundary between strained and unstrained monolayer MoS_2 is presented in Supplementary Figure 4a. The upper area consists of the flat SiO_2 surface and the lower area contains SiO_2 nanocones. A polygon-shaped monolayer MoS_2 (region demarcated by dash lines) covers both flat SiO_2 and nanocones. Scanning Raman images shown in Supplementary Figures 4b-d are recorded simultaneously in the square. Supplementary Figure

4b displays the silicon Raman peak (around 521 cm^{-1}) intensity image. As the nanocones are taller than the flat area, more silicon atoms are in the depth of focus of the Raman objective, resulting in higher Raman peak intensity. As such, the periodic nanocone array can be clearly visualized in the lower part of silicon Raman peak intensity mapping. Supplementary Figures 4c-d show the scanning Raman images of E_{2g}^{1} and A_{1g} peak frequencies of MoS_2, respectively, where bright color indicates higher frequency and dark color represents lower frequency. It is noted that lower frequency appears centered on nanocones while higher frequency appears between nanocones. In contrast, even higher frequency appears on the flat SiO_2 surface. Since tensile strain decreases the Raman peak frequencies of MoS_2, one can see that MoS_2 on nanocones is more strained while that between nanocones is less strained, and that on the flat surface is least strained (or unstrained).

Supplementary Note 4: Scanning tunneling microscopy characterization

Scanning tunneling microscopy and spectroscopy (STM/STS) measurements were performed at 77 K in ultrahigh vacuum (UHV). A bilayer of titanium (10 nm) and gold (80 nm) films were deposited onto the silicon oxide nanocone surface, then monolayer MoS_2 flakes were transferred and strained on the metalized nanocone surface. A control sample was prepared alongside the STM sample and characterized using SEM and AFM to ensure conformal coating of MoS_2 on the metalized nanocones. The STM sample was annealed in the STM UHV chamber at 200 ^{o}C for 1 hour to clean the MoS_2 surface for a reliable STM measurement.

The STS raw $I(V)$ measurements were smoothed using a locally weighted polynomial regression (LOESS) and dI/dV was calculated numerically using a two point central difference. To extract the band gap from dI/dV accurately the curve was first normalized by calculating $(dI/dV)/(I/V)$, where a small positive offset was first applied to I/V to prevent divergence at the band edges[5]. The normalized dI/dV was plotted and first order polynomials were fit to the linear sections of the conduction and valence bands using least-squares regression (Supplementary Figure 5). The polynomial fits were extrapolated to zero to find the band edges and thus the quasiparticle gap size. The example used in Supplementary Figure 5 is the normalized version of one of the single MS-MoS_2 dI/dV data sets plotted in Figure 4b. The normalization process

exaggerates the measurement noise inside the band gap, but this does not affect the gap fitting process since only values outside of the gap are used.

Supplementary Note 5: Modeling and calculations

Modeling of the strain distribution in MoS_2

In order to simulate the MoS_2 geometry on nanocones, we used a Tersoff potential with the parameters presented in previous work (ref. 8 of manuscript). The MoS_2 sheet was modeled using a honeycomb lattice and the substrate with a FCC lattice. The nanocone was modeled as a partial sphere (Supplementary Figure 6a). A Lennard-Jones potential was employed to include the van der Waals interaction between substrate and the MoS_2 sheet as shown in Supplementary Equation 1,

$$V(r) = 4\epsilon\left[\left(\frac{\sigma}{r}\right)^{12} - \left(\frac{\sigma}{r}\right)^{6}\right] \quad (1)$$

where $\epsilon = 0.1$ eV and $\sigma = 6.38$ nm.

A constant force was placed on each atom downward to emulate the pressure difference between top and bottom surfaces of the MoS_2 sheet. The periodic boundary condition was employed in x and y directions. The interaction (between substrate and MoS_2 sheet) and the pressure were tuned so that the trace of strain on tip of the cone matches the value obtained from experiment. In order to calculate half of the trace of strain, we took advantage of the fact that the change in the area of the surface is the same as the trace of strain. A simulated annealing was used to find the global minimum. The final minimum was achieved with conjugate gradient. The resultant strain was fitted using Fourier series. The 12-fold symmetry of the problem is illustrated in Supplementary Figure 6b, and the final local biaxial strain distribution is plotted in Supplementary Figure 6c.

Theoretical optical absorption spectra of strained monolayer MoS_2

The theoretical biaxial-strain-dependent optical absorption spectra were calculated by solving the Bethe-Salpeter equation[6] within the Tamm–Dancoff approximation. The key parameters used in the Bethe-Salpeter equation, including quasiparticle energies and screened-

Coulomb interactions, were obtained from many-body perturbation theory with the Hedin's GW approximation[7-9]. All the calculations were performed using the Vienna Ab initio Simulation Package (VASP)[10,11] with plane-wave basis and the projector-augmented wave (PAW) method[12]. We used a plane-wave cutoff of 350 eV, a Monkhorst–Pack **k**-point sampling[13] of 18×18×1, and an exchange correlation functional of the Perdew–Berke–Ernzerhof form[14] within the generalized gradient approximation[15,16]. All biaxially-strained configuration were fully relaxed with the maximal residual force of no more than 0.0001 eV/Å using density-functional theory calculations[17,18]. The calculation was carried out in a periodic supercell with a vacuum spacing of 20 Å along the z (plane normal) direction in order to reduce the spurious interaction between the neighboring unit cells. Although spin-orbit coupling was not included in the present calculations, the peak position and the strain-dependent peak shift of the first low energy exciton were expected to be minimal. The calculated optical absorption spectra of MoS_2 under average biaxial tensile strain $\tilde{\varepsilon}_{\mathrm{bi}}$ from 0 to 1% are presented in Supplementary Figure 7a.

Theoretical Raman spectra of strained monolayer MoS_2

The theoretical biaxial-strain-dependent Raman spectra were calculated using first-principles density-functional perturbation theory implemented in the QUATUM-ESPRESSO package[19,20] with a plane-wave cutoff of 120 Rydberg, a Monkhorst–Pack **k**-point sampling of 12×12×1, and an exchange correlation functional of the Perdew-Zunger[21] form within the local density approximation. The spin-orbit coupling was not included. In addition, norm-conserving Hartwigsen-Goedecker-Hutter pseudopotentials[22] were used in order to take into account the core electrons and reduce the computational efforts. All biaxially-strained configuration were fully relaxed with a convergence criteria of 0.0001 a.u. for the maximal residual force. The calculation was carried out in a periodic supercell with a vacuum spacing of 20 Å along the z (plane normal) direction in order to reduce the spurious interaction between the neighboring unit cells. At the long wavelength limit, the degeneracy between longitudinal and transverse optical phonons is lifted due to the macroscopic electric field induced by infrared-active phonon modes, which leads to a splitting in the E_{2g}^{1} mode into the transverse optical (TO) and longitudinal optical (LO) modes, respectively[23]. For simplicity, we plot the average frequency of TO- and LO-E_{2g}^{1} modes in the manuscript. The calculated Raman spectra of monolayer MoS_2 under average biaxial tensile strain $\tilde{\varepsilon}_{\mathrm{bi}}$ from 0% to 1% are shown in Supplementary Figure 7b.

Supplementary Note 6: Optimization of dimension and geometry of nanostructured substrate for straining MoS_2

Since nanospheres were used as the etch mask, round-tip nanocones instead of tapered-nanopillars can be obtained with appropriate etching processes. The shape of the nanostructure is important for obtaining regular and uniform strain profiles. Supplementary Figure 8a summarizes the measured optically averaged strain $\tilde{\varepsilon}_{\mathrm{bi}}$ (derived from $E_{2\mathrm{g}}^{1}$ Raman peak shift) at different nanocone heights (diameter of nanosphere mask is fixed at D = 500 nm). Regular wrinkles arising from strain in MoS_2 can be observed when the nanocone is taller than 30 nm, where the optically averaged biaxial strain magnitude is about 0.22%. The strain magnitude increases with increasing nanocone height (H). However, the strain distribution becomes nonuniform when H is larger than 90 nm due to suspending MoS_2 in some areas [see Supplementary Figure 8d(II)] because the adhesion (van der Waals interaction) between MoS_2 and substrate is not large enough to balance the restoring force arising from the large strain in MoS_2 (see Supplementary Figure 8b for strain distribution at H = 120 nm). When H is larger than 500 nm, MoS_2 would suspend on the tip of the nanopillars without touching the valley, similar to graphene (ref. 26 of manuscript) where the average strain is about 0.15%. It is noted that the shape of the nanocone also varies when H changes. The irregular shape of nanocones may also contribute to the nonuniformity of strain distribution.

Only larger spheres (D > 500 nm) can be studied due to the limited spatial resolution of Raman/PL spectroscopy. Supplementary Figure 8c presents the scanning Raman image of peak $E_{2\mathrm{g}}^{1}$ frequency of MoS_2 strained using nanopillar (H = 90 nm and D = 1000 nm). No strain gradient can be observed from perimeter to the center of the nanopillar that has a flat top surface [see Supplementary Figure 8d(III)]. Considering both factors of dimension and geometry, H = 90 nm and D = 500 nm (round-tip nanocone) offer the optimized conditions to achieve the strain distribution for an "artificial atom." With optimization, we also minimize the film breaking and more than 70% of the MoS_2 sheet remains intact after transfer process.

Supplementary References:

1. Najmaei, S. *et al.*, Vapour phase growth and grain boundary structure of molybdenum disulphide atomic layers. *Nat. Mater.* **12**, 754-759 (2013).

2. Tas, N. R., Mela, P., Kramer, T., Berenschot, J. W. & Berg, A. van den. Capillarity Induced Negative Pressure of Water Plugs in Nanochannels. *Nano Lett.* **3**, 1537-1540 (2003).

3. van Honschoten, J. W., Brunets, N. & Tas, N. R. Capillarity at the nanoscale. *Chem. Sci. Rev.* **39**, 1096-1114 (2010).

4. Rafati, A. A., Ghasemian, E. & Abdolmaleki, M. Surface Properties of Binary Mixtures of Ethylene Glycol with a Series of Aliphatic Alcohols (1-Pentanol, 1-Hexanol, and 1-Heptanol). *J. Chem. Eng. Data* **53**, 1944-1949 (2008).

5. Stroscio, J. A. & Feenstra, R. M. in *Scanning Tunneling Microscopy*, vol. 27 of Methods in Experimental Physics, J. A. Stroscio, W. J. Kaiser (Academic Press, San Diego, 1993), 138-140.

6. Salpeter, E. E. & Bethe, H. A. A relativistic equation for bound-state problems. *Phys. Rev.* **84**, 1232-1242 (1951).

7. Hedin, L. New method for calculating one-particle Green's function with application to electron-gas problem. *Phys. Rev.* **139**, A796-A823 (1965).

8. Hybertsen, M. S. & Louie, S. G. First-principles theory of quasiparticles: calculation of band-gaps in semiconductors and insulators. *Phys. Rev. Lett.* **55**, 1418-1421 (1985).

9. Onida, G. & Reining, L. & Rubio, A. Electronic excitations: density-functional versus many-body Green's-function approaches. *Rev. Mod. Phys.* **74**, 601-659 (2002).

10. Kresse, G. & Furthmuller, J. Efficiency of ab-initio total energy calculations for metals and semiconductors using a plane-wave basis set. *Comput. Mat. Sci.* **6**, 15-50 (1996).

11. Kresse, G. & Furthmuller, J. Efficient iterative schemes for ab initio total-energy calculations using a plane-wave basis set. *Phys. Rev. B* **54**, 11169-11186 (1996).

12. Blöchl, P. E. Projector augmented-wave method. *Phys. Rev. B* **50**, 17953-17979 (1994).

13. Monkhorst, H. J. & Pack, J. D. Special points for brillouin-zone integrations. *Phys. Rev. B* **13**, 5188-5192 (1976).

14. Perdew, J. P., Burke, K. & Ernzerhof, M. Generalized gradient approximation made simple. *Phys. Rev. Lett.* **77**, 3865-3868 (1996).

15. Langreth, D. C. & Mehl, M. J. Beyond the local-density approximation in calculations of ground-state electronic-properties. *Phys. Rev. B* **28**, 1809-1834 (1983).

16. Becke, A. D. Density-functional exchange-energy approximation with correct asymptotic-behavior. *Phys. Rev. A* **38**, 3098-3100 (1988).

17. Hohenberg, P. & Kohn, W. Inhomogeneous electron gas. *Phys. Rev. B* **136**, B864-B871 (1964).

18. Kohn, W. & Sham, L. J. Self-consistent equations including exchange and correlation effects. *Phys. Rev.* **140**, A1133-A1138 (1965).

19. Baroni, S., Gironcoli, S. de, Corso, A. Dal & Giannozzi, P. Phonons and related crystal properties from density-functional perturbation theory. *Rev. Mod. Phys.* **73**, 515-562 (2001).

20. Giannozzi, P. *et al.*, QUANTUM ESPRESSO: a modular and open-source software project for quantum simulations of materials. *J. Phys.: Condens. Matter* **21**, 395502 (2009).

21. Perdew, J. P. & Zunger, A. Self-interaction correction to density-functional approximations for many-electron systems. *Phys. Rev. B* **23**, 5048-5079 (1981).

22. Hartwigsen, C., Goedecker, S. & Hutter, J. Relativistic separable dual-space Gaussian pseudopotentials from H to Rn. *Phys. Rev. B* **58**, 3641-3662 (1998).

23. Molina-Sánchez, A. & Wirtz, L. Phonons in single-layer and few-layer MoS_2 and WS_2. *Phys. Rev. B* **84**, 155413 (2011).
